# Ethyl 2-{[(1*Z*)-(3-methyl-5-oxo-1-phenyl-4,5-dihydro-1*H*-pyrazol-4-yl­idene)(*p*-tol­yl)meth­yl]amino}-3-phenyl­propanoate

**DOI:** 10.1107/S1600536809054865

**Published:** 2010-01-09

**Authors:** Xin Zhang, Meng Huang, Cong Du, Junjing Han

**Affiliations:** a College of Chemistry and Life Science, Tianjin Normal University, Tianjin 300387, People’s Republic of China

## Abstract

The asymmetric unit of the title compound, C_29_H_29_N_3_O_3_, contains two mol­ecules, which exist in their enamine–keto form, being stabilized by strong intra­molecular N—H⋯O hydrogen bonds, which generate *S*(6) loops. In the crystal, inter­molecular C–H⋯O hydrogen bonds link the mol­ecules into chains, which are further linked by weak C—H⋯π inter­actions, forming a two-dimensional network.

## Related literature

For general background to Schiff base compounds in coordination chemistry, see: Habibi *et al.* (2007 ▶). For the anti­bacterial properties of Schiff bases derived from 4-acyl-5-pyrazolones and their metal complexes, see: Li *et al.* (1997 ▶, 2004 ▶). For the anti­bacterial and biological activity of amino acid esters, see: Xiong *et al.* (1993 ▶). For related structures, see: Wang *et al.* (2003 ▶); Zhang *et al.* (2004 ▶). For further synthetic details, see: Remya *et al.* (2005 ▶).
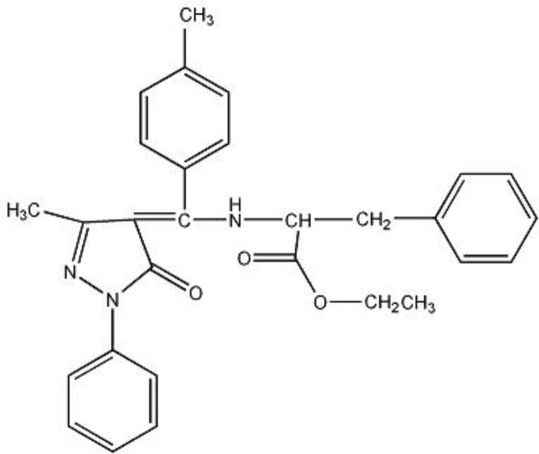


## Experimental

### 

#### Crystal data

C_29_H_29_N_3_O_3_
*M* *_r_* = 467.55
Triclinic, 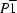

*a* = 11.0637 (11) Å
*b* = 13.2746 (14) Å
*c* = 20.299 (2) Å
α = 101.869 (2)°
β = 97.923 (2)°
γ = 112.861 (2)°
*V* = 2608.8 (5) Å^3^
*Z* = 4
Mo *K*α radiation
μ = 0.08 mm^−1^
*T* = 296 K
0.38 × 0.32 × 0.26 mm

#### Data collection

Bruker SMART CCD diffractometer
Absorption correction: multi-scan (*SADABS*; Bruker, 1999 ▶) *T*_min_ = 0.971, *T*_max_ = 0.980
13503 measured reflections
9153 independent reflections
5450 reflections with *I* > 2σ(*I*)
*R*_int_ = 0.021

#### Refinement

*R*[*F* ^2^ > 2σ(*F* ^2^)] = 0.048
*wR*(*F* ^2^) = 0.132
*S* = 1.01
9153 reflections
636 parameters
H-atom parameters constrained
Δρ_max_ = 0.18 e Å^−3^
Δρ_min_ = −0.19 e Å^−3^

### 

Data collection: *SMART* (Bruker, 1999 ▶); cell refinement: *SAINT* (Bruker, 1999 ▶); data reduction: *SAINT*; program(s) used to solve structure: *SHELXS97* (Sheldrick, 2008 ▶); program(s) used to refine structure: *SHELXL97* (Sheldrick, 2008 ▶); molecular graphics: *SHELXTL* (Sheldrick, 2008 ▶); software used to prepare material for publication: *SHELXTL*.

## Supplementary Material

Crystal structure: contains datablocks I, global. DOI: 10.1107/S1600536809054865/hb5268sup1.cif

Structure factors: contains datablocks I. DOI: 10.1107/S1600536809054865/hb5268Isup2.hkl

Additional supplementary materials:  crystallographic information; 3D view; checkCIF report

## Figures and Tables

**Table 1:** Hydrogen-bond geometry (Å, °) *Cg*6 is the centroid of C30–C35 ring.

*D*—H⋯*A*	*D*—H	H⋯*A*	*D*⋯*A*	*D*—H⋯*A*
N3—H3*A*⋯O1	0.86	2.01	2.713 (2)	138
N6—H6⋯O4	0.86	2.03	2.698 (2)	133
C16—H16⋯O6^i^	0.93	2.51	3.397 (3)	161
C28—H28*B*⋯O1^ii^	0.97	2.44	3.357 (3)	157
C45—H45⋯*Cg*6^iii^	0.93	2.77	3.449 (3)	130
C57—H57*B*⋯*Cg*6^iv^	0.96	2.78	3.663 (3)	152

Symmetry codes: (i) 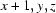; (ii) 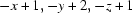; (iii) 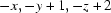; (iv) 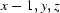.

**Fig. 1.**
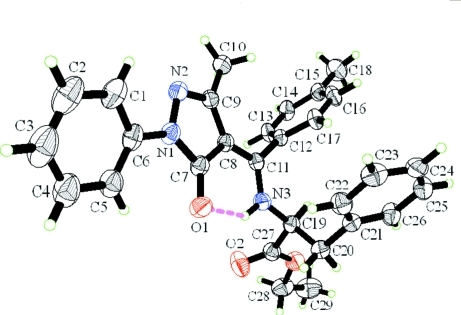
A view of (I), showing displacement ellipsoids drawn at the 30% probability level. The intra-molecular hydrogen bond is indicated by dashed line.

**Fig. 2.**
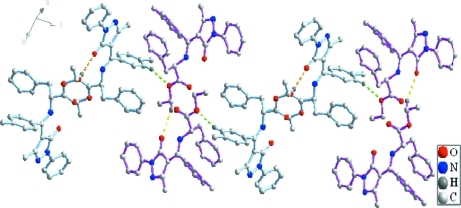
The one-dimensional plane of (I) formed by the intermolecular C–H···O weak hydrogen-bonding interactions.

**Fig. 3.**
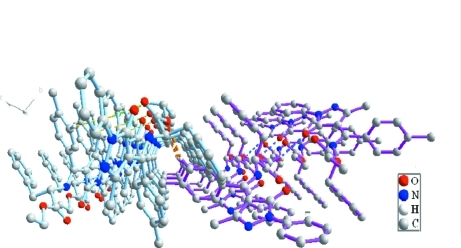
The two-dimensional supra-molecular network of (I) produced by the inter-molecular C–H ···π interactions.

## supplementary crystallographic information

### Comment

In recent years, Schiff base compounds play an important role in the development of coordination chemistry related to catalysis and enzymatic reactions, magnetism, and molecular architectures (Habibi *et al.*, 2007). The structures of Schiff bases derived from 4-acyl-5-pyrazolones and their metal complexes have been studied widely for their high antibacterial activation (Li *et al.*, 1997, 2004). Both 1-phenyl-3-methyl-4-toluoyl-5-pyrazolone, Hpmtp, and its metal complexes are widely used and well known for their analgesic activity (Remya *et al.*, 2005). Since amino acid esters also possess good antibacterial and biological activations (Xiong *et al.*, 1993), we have studied the reactions of Hpmtp and amino acid esters.

In the molecule of the title compound, (I), (Fig.1) there are two molecules in the asymmetric unit, and the numerical results given here are for one of them; they are not significantly different. Atoms O1, C7, C8, C11 and N3 form a plane, the largest deviation being 0.0310 Å for atom C11. The dihedral angle between this mean plane and the pyrazolone ring is 1.52 (4)°, indicating that they are essentially coplanar, as seen in 4-{[3,4-dihydro-5-methyl-3-oxo-2-phenyl-2*H*-pyrazol-4-ylidene]-(phenyl) methyl] amino}-1,5-dimethyl-2-phenyl-1*H*-pyrazol-3(2*H*)-one(3.56 (3)°;Wang *et al.*,2003). The bond lengths within this part of the molecule lie between classical single- and double-bond lengths, indicating extensive conjugation. Atoms N3, C19, C27 and O2 are not coplanar, the torsion angle being 22.5 (3)°, different from that in some other 4-acyl-5-prazolone Schiff bases (Zhang *et al.*, 2004; Wang *et al.*, 2003). The bond lengths in this part of the molecule indicate that only C27=O2 is a classical double bond and the other bonds are classical single bonds. The dihedral angle between the benzene ring of ethyl 2-amino-3-phenyl-propanoate and the pyrazolone ring is 63.95 (2)°, reducing steric hindrance. A strong intramolecular N–H···O hydrogen bond is observed (Table 1), stabilizing the enamine-keto form.

In the crystal structure, inter-molecular C–H···O hydrogen bonds (Table 1) link the molecules into 1-D chain (Fig.2), which is further linked by weak C–H···π interactions (Table 1) to form a two-dimensional network (Fig.3), in which they may be effective in the stabilization of the structure.

### Experimental

The title compound was synthesized by refluxing a mixture of 1-phenyl-3-methyl-4-toluoyl-5-pyrazolone (15 mmol) (Remya *et al.*, 2005) and phenylalanine ethyl ester (15 mmol) in ethanol (100 ml) for about 5 h. The product was recrystallized from ethanol, affording pale yellow crystals suitable for X-ray analysis.

### Refinement

All H atoms were positioned geometrically (N–H = 0.86Å and C–H = 0.93–0.97Å) and treated as riding, with *U*_iso_(H)= 1.2*U*_eq_(C); the *U*_iso_ value for the H atoms bonded to N atoms was refined freely. The ethyl group was found to be disordered and was refined as two components with equal occupancy, with the acid of restraints on geometry and displacement parameters.

### Crystal data

**Table:**

C_29_H_29_N_3_O_3_	*Z* = 4
*M**_r_* = 467.55	*F*(000) = 992
Triclinic, *P*1	*D*_x_ = 1.190 Mg m^−^^3^
Hall symbol: -P 1	Mo *K*α radiation, λ = 0.71073 Å
*a* = 11.0637 (11) Å	Cell parameters from 2516 reflections
*b* = 13.2746 (14) Å	θ = 2.3–20.2°
*c* = 20.299 (2) Å	µ = 0.08 mm^−^^1^
α = 101.869 (2)°	*T* = 296 K
β = 97.923 (2)°	Block, yellow
γ = 112.861 (2)°	0.38 × 0.32 × 0.26 mm
*V* = 2608.8 (5) Å^3^	

### Data collection

**Table:**

Bruker SMART CCD diffractometer	9153 independent reflections
Radiation source: fine-focus sealed tube	5450 reflections with *I* > 2σ(*I*)
graphite	*R*_int_ = 0.021
ω scans	θ_max_ = 25.0°, θ_min_ = 1.7°
Absorption correction: multi-scan (*SADABS*; Bruker, 1999)	*h* = −13→11
*T*_min_ = 0.971, *T*_max_ = 0.980	*k* = −10→15
13503 measured reflections	*l* = −24→23

### Refinement

**Table:**

Refinement on *F*^2^	Primary atom site location: structure-invariant direct methods
Least-squares matrix: full	Secondary atom site location: difference Fourier map
*R*[*F*^2^ > 2σ(*F*^2^)] = 0.048	Hydrogen site location: inferred from neighbouring sites
*wR*(*F*^2^) = 0.132	H-atom parameters constrained
*S* = 1.01	*w* = 1/[σ^2^(*F*_o_^2^) + (0.0568*P*)^2^ + 0.1678*P*] where *P* = (*F*_o_^2^ + 2*F*_c_^2^)/3
9153 reflections	(Δ/σ)_max_ = 0.001
636 parameters	Δρ_max_ = 0.18 e Å^−^^3^
0 restraints	Δρ_min_ = −0.19 e Å^−^^3^

### Special details

**Table:**

Geometry. All e.s.d.'s (except the e.s.d. in the dihedral angle between two l.s. planes) are estimated using the full covariance matrix. The cell e.s.d.'s are taken into account individually in the estimation of e.s.d.'s in distances, angles and torsion angles; correlations between e.s.d.'s in cell parameters are only used when they are defined by crystal symmetry. An approximate (isotropic) treatment of cell e.s.d.'s is used for estimating e.s.d.'s involving l.s. planes.
Refinement. Refinement of *F*^2^ against ALL reflections. The weighted *R*-factor *wR* and goodness of fit *S* are based on *F*^2^, conventional *R*-factors *R* are based on *F*, with *F* set to zero for negative *F*^2^. The threshold expression of *F*^2^ > σ(*F*^2^) is used only for calculating *R*-factors(gt) *etc*. and is not relevant to the choice of reflections for refinement. *R*-factors based on *F*^2^ are statistically about twice as large as those based on *F*, and *R*- factors based on ALL data will be even larger.

### Fractional atomic coordinates and isotropic or equivalent isotropic displacement parameters (Å^2^)

**Table:**

	*x*	*y*	*z*	*U*_iso_*/*U*_eq_	
O1	0.71923 (17)	0.81845 (14)	0.49334 (9)	0.0766 (5)	
O2	0.3638 (2)	0.8629 (2)	0.49767 (10)	0.1062 (7)	
O3	0.29842 (17)	0.92431 (15)	0.59120 (9)	0.0799 (5)	
O4	0.24307 (14)	0.80519 (12)	1.00473 (7)	0.0631 (4)	
O5	−0.08684 (17)	0.87650 (15)	1.01365 (8)	0.0776 (5)	
O6	−0.25145 (15)	0.84962 (13)	0.92501 (7)	0.0649 (4)	
N1	0.77524 (18)	0.66606 (16)	0.49565 (9)	0.0609 (5)	
N2	0.74399 (19)	0.57817 (16)	0.52761 (9)	0.0616 (5)	
N3	0.55387 (18)	0.82573 (16)	0.57946 (9)	0.0617 (5)	
H3A	0.5857	0.8429	0.5449	0.074*	
N4	0.29698 (16)	0.65042 (14)	0.99683 (9)	0.0513 (4)	
N5	0.23374 (18)	0.53247 (14)	0.96488 (9)	0.0547 (5)	
N6	−0.01134 (18)	0.73294 (15)	0.92980 (9)	0.0598 (5)	
H6	0.0499	0.7785	0.9666	0.072*	
C1	0.9533 (3)	0.6259 (2)	0.45793 (15)	0.0834 (8)	
H1	0.9543	0.5868	0.4910	0.100*	
C2	1.0407 (3)	0.6352 (3)	0.4136 (2)	0.1111 (11)	
H2	1.1001	0.6016	0.4171	0.133*	
C3	1.0397 (4)	0.6932 (3)	0.3653 (2)	0.1202 (13)	
H3	1.0988	0.6993	0.3364	0.144*	
C4	0.9522 (3)	0.7420 (3)	0.35953 (17)	0.1045 (10)	
H4	0.9515	0.7809	0.3263	0.125*	
C5	0.8643 (3)	0.7342 (2)	0.40263 (14)	0.0801 (8)	
H5	0.8050	0.7679	0.3987	0.096*	
C6	0.8655 (2)	0.6759 (2)	0.45169 (13)	0.0667 (7)	
C7	0.7118 (2)	0.7346 (2)	0.51423 (11)	0.0591 (6)	
C8	0.6389 (2)	0.68835 (18)	0.56273 (10)	0.0520 (5)	
C9	0.6635 (2)	0.59172 (19)	0.56667 (11)	0.0551 (6)	
C10	0.6086 (3)	0.5064 (2)	0.60524 (12)	0.0732 (7)	
H10A	0.6391	0.4479	0.5940	0.110*	
H10B	0.5116	0.4729	0.5923	0.110*	
H10C	0.6397	0.5435	0.6542	0.110*	
C11	0.5662 (2)	0.73839 (18)	0.59647 (10)	0.0508 (5)	
C12	0.5048 (2)	0.69619 (18)	0.65205 (10)	0.0497 (5)	
C13	0.3756 (2)	0.6121 (2)	0.63623 (11)	0.0634 (6)	
H13	0.3223	0.5857	0.5912	0.076*	
C14	0.3246 (3)	0.5665 (2)	0.68735 (12)	0.0720 (7)	
H14	0.2368	0.5095	0.6760	0.086*	
C15	0.3998 (3)	0.6030 (2)	0.75424 (12)	0.0689 (7)	
C16	0.5282 (3)	0.6890 (2)	0.76938 (12)	0.0757 (7)	
H16	0.5810	0.7159	0.8145	0.091*	
C17	0.5807 (2)	0.7364 (2)	0.71958 (11)	0.0648 (6)	
H17	0.6672	0.7954	0.7315	0.078*	
C18	0.3415 (3)	0.5521 (3)	0.80965 (15)	0.1137 (11)	
H18A	0.2749	0.5776	0.8210	0.170*	
H18B	0.4126	0.5760	0.8504	0.170*	
H18C	0.3003	0.4704	0.7925	0.170*	
C19	0.4935 (2)	0.89585 (19)	0.61186 (11)	0.0596 (6)	
H19	0.4596	0.8685	0.6500	0.071*	
C20	0.5985 (2)	1.0210 (2)	0.64057 (12)	0.0695 (7)	
H20A	0.6400	1.0446	0.6039	0.083*	
H20B	0.5525	1.0681	0.6543	0.083*	
C21	0.7078 (2)	1.04170 (19)	0.70174 (12)	0.0595 (6)	
C22	0.8205 (2)	1.0246 (2)	0.69331 (13)	0.0709 (7)	
H22	0.8304	0.9999	0.6489	0.085*	
C23	0.9190 (3)	1.0439 (2)	0.75043 (16)	0.0812 (8)	
H23	0.9946	1.0320	0.7443	0.097*	
C24	0.9055 (3)	1.0806 (2)	0.81618 (15)	0.0781 (8)	
H24	0.9723	1.0943	0.8545	0.094*	
C25	0.7943 (3)	1.0970 (2)	0.82516 (14)	0.0806 (8)	
H25	0.7846	1.1215	0.8696	0.097*	
C26	0.6963 (3)	1.0772 (2)	0.76821 (13)	0.0758 (7)	
H26	0.6202	1.0880	0.7748	0.091*	
C27	0.3774 (3)	0.8892 (2)	0.55943 (14)	0.0695 (7)	
C28	0.1891 (3)	0.9351 (3)	0.54875 (16)	0.0943 (9)	
H28A	0.1342	0.8644	0.5125	0.113*	
H28B	0.2257	0.9955	0.5271	0.113*	
C29	0.1067 (3)	0.9618 (3)	0.59471 (19)	0.1302 (13)	
H29A	0.0646	0.8986	0.6126	0.195*	
H29B	0.0384	0.9759	0.5690	0.195*	
H29C	0.1636	1.0284	0.6325	0.195*	
C30	0.4752 (2)	0.7986 (2)	1.09284 (12)	0.0640 (6)	
H30	0.4203	0.8358	1.1002	0.077*	
C31	0.6034 (3)	0.8419 (2)	1.13540 (14)	0.0793 (7)	
H31	0.6349	0.9087	1.1714	0.095*	
C32	0.6846 (3)	0.7867 (3)	1.12470 (16)	0.0868 (8)	
H32	0.7700	0.8153	1.1541	0.104*	
C33	0.6400 (3)	0.6900 (3)	1.07104 (16)	0.0837 (8)	
H33	0.6958	0.6537	1.0633	0.100*	
C34	0.5129 (2)	0.6461 (2)	1.02831 (13)	0.0667 (6)	
H34	0.4828	0.5801	0.9918	0.080*	
C35	0.4297 (2)	0.70014 (18)	1.03960 (11)	0.0527 (5)	
C36	0.2138 (2)	0.70135 (18)	0.98212 (10)	0.0486 (5)	
C37	0.0921 (2)	0.61086 (17)	0.93655 (10)	0.0472 (5)	
C38	0.1131 (2)	0.50964 (17)	0.92975 (10)	0.0503 (5)	
C39	0.0195 (2)	0.38911 (18)	0.89275 (12)	0.0700 (7)	
H39A	0.0112	0.3770	0.8437	0.105*	
H39B	−0.0676	0.3725	0.9024	0.105*	
H39C	0.0544	0.3399	0.9082	0.105*	
C40	−0.0169 (2)	0.62915 (18)	0.90837 (10)	0.0486 (5)	
C41	−0.1392 (2)	0.54022 (17)	0.85620 (10)	0.0490 (5)	
C42	−0.1335 (2)	0.4956 (2)	0.78968 (11)	0.0642 (6)	
H42	−0.0510	0.5193	0.7775	0.077*	
C43	−0.2494 (3)	0.4158 (2)	0.74106 (12)	0.0730 (7)	
H43	−0.2436	0.3865	0.6965	0.088*	
C44	−0.3731 (3)	0.3787 (2)	0.75699 (13)	0.0706 (7)	
C45	−0.3777 (2)	0.4225 (2)	0.82360 (14)	0.0722 (7)	
H45	−0.4601	0.3975	0.8358	0.087*	
C46	−0.2635 (2)	0.5025 (2)	0.87262 (12)	0.0620 (6)	
H46	−0.2698	0.5314	0.9171	0.074*	
C47	−0.5008 (3)	0.2919 (2)	0.70330 (15)	0.1088 (11)	
H47A	−0.4778	0.2659	0.6612	0.163*	
H47B	−0.5607	0.3265	0.6942	0.163*	
H47C	−0.5448	0.2283	0.7206	0.163*	
C48	−0.0969 (2)	0.77755 (18)	0.89797 (11)	0.0546 (6)	
H48	−0.1760	0.7146	0.8644	0.065*	
C49	−0.0201 (2)	0.86357 (19)	0.86083 (12)	0.0654 (6)	
H49A	−0.0807	0.8914	0.8401	0.078*	
H49B	0.0539	0.9282	0.8950	0.078*	
C50	0.0360 (2)	0.81547 (19)	0.80509 (12)	0.0611 (6)	
C51	0.1668 (3)	0.8278 (2)	0.81830 (15)	0.0867 (8)	
H51	0.2226	0.8663	0.8626	0.104*	
C52	0.2163 (3)	0.7838 (3)	0.76661 (19)	0.1075 (10)	
H52	0.3050	0.7924	0.7764	0.129*	
C53	0.1357 (4)	0.7275 (3)	0.70104 (17)	0.1027 (10)	
H53	0.1699	0.6993	0.6660	0.123*	
C54	0.0062 (3)	0.7131 (3)	0.68762 (14)	0.0938 (9)	
H54	−0.0496	0.6736	0.6434	0.113*	
C55	−0.0432 (3)	0.7568 (2)	0.73906 (13)	0.0792 (7)	
H55	−0.1326	0.7464	0.7290	0.095*	
C56	−0.1432 (2)	0.83904 (18)	0.95348 (12)	0.0559 (6)	
C57	−0.3000 (2)	0.9187 (2)	0.96945 (12)	0.0712 (7)	
H57A	−0.2362	0.9984	0.9827	0.085*	
H57B	−0.3104	0.8949	1.0112	0.085*	
C58	−0.4329 (3)	0.9022 (2)	0.92927 (14)	0.0898 (8)	
H58A	−0.4228	0.9202	0.8863	0.135*	
H58B	−0.4643	0.9516	0.9558	0.135*	
H58C	−0.4973	0.8244	0.9199	0.135*	

### Atomic displacement parameters (Å^2^)

**Table:**

	*U*^11^	*U*^22^	*U*^33^	*U*^12^	*U*^13^	*U*^23^
O1	0.0967 (13)	0.0666 (11)	0.0890 (12)	0.0408 (10)	0.0529 (10)	0.0364 (10)
O2	0.1053 (15)	0.157 (2)	0.0633 (12)	0.0714 (15)	0.0118 (11)	0.0198 (13)
O3	0.0790 (12)	0.0933 (13)	0.0854 (12)	0.0542 (11)	0.0211 (10)	0.0268 (10)
O4	0.0664 (10)	0.0462 (9)	0.0731 (10)	0.0302 (8)	0.0019 (8)	0.0076 (8)
O5	0.0844 (12)	0.0958 (13)	0.0531 (10)	0.0533 (11)	0.0010 (9)	0.0034 (9)
O6	0.0677 (10)	0.0708 (11)	0.0603 (9)	0.0456 (9)	0.0032 (8)	0.0033 (8)
N1	0.0631 (12)	0.0593 (12)	0.0646 (12)	0.0269 (10)	0.0288 (10)	0.0165 (10)
N2	0.0683 (12)	0.0587 (12)	0.0589 (11)	0.0290 (10)	0.0182 (10)	0.0138 (10)
N3	0.0771 (13)	0.0679 (13)	0.0563 (11)	0.0393 (11)	0.0305 (10)	0.0248 (10)
N4	0.0500 (11)	0.0447 (11)	0.0613 (11)	0.0261 (9)	0.0077 (9)	0.0117 (9)
N5	0.0607 (12)	0.0452 (11)	0.0615 (11)	0.0280 (9)	0.0113 (10)	0.0136 (9)
N6	0.0675 (12)	0.0537 (12)	0.0559 (11)	0.0363 (10)	−0.0058 (9)	0.0042 (9)
C1	0.0691 (17)	0.0748 (18)	0.099 (2)	0.0304 (15)	0.0289 (16)	0.0060 (15)
C2	0.083 (2)	0.091 (2)	0.155 (3)	0.0365 (19)	0.059 (2)	0.008 (2)
C3	0.110 (3)	0.091 (3)	0.157 (3)	0.028 (2)	0.092 (3)	0.021 (2)
C4	0.120 (3)	0.084 (2)	0.118 (2)	0.035 (2)	0.078 (2)	0.0294 (19)
C5	0.0825 (19)	0.0724 (18)	0.0863 (18)	0.0255 (15)	0.0465 (16)	0.0224 (15)
C6	0.0580 (15)	0.0572 (15)	0.0730 (16)	0.0168 (12)	0.0272 (13)	0.0028 (13)
C7	0.0584 (14)	0.0550 (15)	0.0615 (14)	0.0218 (12)	0.0206 (12)	0.0124 (12)
C8	0.0537 (13)	0.0542 (13)	0.0488 (12)	0.0230 (11)	0.0177 (10)	0.0131 (11)
C9	0.0577 (14)	0.0597 (15)	0.0465 (12)	0.0260 (12)	0.0109 (11)	0.0118 (11)
C10	0.0963 (19)	0.0688 (17)	0.0644 (15)	0.0409 (15)	0.0259 (14)	0.0242 (13)
C11	0.0477 (12)	0.0528 (14)	0.0436 (12)	0.0165 (11)	0.0078 (10)	0.0088 (10)
C12	0.0508 (13)	0.0546 (13)	0.0423 (12)	0.0233 (11)	0.0123 (10)	0.0088 (10)
C13	0.0614 (15)	0.0665 (16)	0.0465 (13)	0.0158 (13)	0.0101 (11)	0.0093 (12)
C14	0.0735 (16)	0.0625 (16)	0.0628 (16)	0.0111 (13)	0.0263 (14)	0.0123 (13)
C15	0.0896 (19)	0.0717 (17)	0.0528 (15)	0.0370 (16)	0.0308 (14)	0.0194 (13)
C16	0.0830 (19)	0.104 (2)	0.0425 (13)	0.0455 (18)	0.0106 (13)	0.0170 (14)
C17	0.0558 (14)	0.0799 (17)	0.0482 (14)	0.0245 (13)	0.0083 (11)	0.0087 (13)
C18	0.155 (3)	0.123 (3)	0.088 (2)	0.060 (2)	0.066 (2)	0.056 (2)
C19	0.0682 (15)	0.0646 (15)	0.0545 (13)	0.0357 (13)	0.0191 (12)	0.0175 (12)
C20	0.0818 (17)	0.0656 (16)	0.0653 (15)	0.0384 (14)	0.0125 (13)	0.0174 (13)
C21	0.0676 (15)	0.0531 (14)	0.0584 (15)	0.0268 (12)	0.0148 (12)	0.0157 (11)
C22	0.0697 (17)	0.0745 (17)	0.0682 (16)	0.0282 (14)	0.0213 (14)	0.0224 (13)
C23	0.0619 (17)	0.0771 (19)	0.103 (2)	0.0280 (15)	0.0158 (16)	0.0279 (17)
C24	0.082 (2)	0.0573 (16)	0.0770 (19)	0.0183 (14)	−0.0039 (15)	0.0207 (14)
C25	0.094 (2)	0.0754 (19)	0.0606 (16)	0.0312 (17)	0.0121 (16)	0.0096 (14)
C26	0.0777 (17)	0.0802 (19)	0.0651 (17)	0.0374 (15)	0.0150 (14)	0.0062 (14)
C27	0.0703 (17)	0.0749 (18)	0.0681 (18)	0.0339 (15)	0.0199 (14)	0.0227 (14)
C28	0.0720 (18)	0.093 (2)	0.121 (2)	0.0429 (17)	0.0049 (18)	0.0337 (19)
C29	0.110 (3)	0.145 (3)	0.185 (4)	0.088 (3)	0.056 (3)	0.069 (3)
C30	0.0568 (15)	0.0622 (16)	0.0718 (16)	0.0288 (13)	0.0098 (13)	0.0141 (13)
C31	0.0630 (17)	0.0732 (18)	0.0826 (18)	0.0192 (15)	0.0029 (14)	0.0134 (14)
C32	0.0466 (15)	0.092 (2)	0.109 (2)	0.0200 (16)	0.0009 (15)	0.0339 (19)
C33	0.0530 (16)	0.086 (2)	0.118 (2)	0.0347 (15)	0.0158 (16)	0.0331 (19)
C34	0.0590 (15)	0.0660 (16)	0.0834 (17)	0.0333 (13)	0.0197 (13)	0.0236 (13)
C35	0.0488 (13)	0.0527 (14)	0.0617 (14)	0.0247 (11)	0.0125 (11)	0.0216 (12)
C36	0.0538 (13)	0.0473 (13)	0.0527 (13)	0.0282 (11)	0.0133 (10)	0.0172 (11)
C37	0.0514 (13)	0.0460 (12)	0.0505 (12)	0.0281 (11)	0.0103 (10)	0.0139 (10)
C38	0.0570 (14)	0.0468 (13)	0.0497 (12)	0.0252 (11)	0.0112 (11)	0.0146 (10)
C39	0.0757 (16)	0.0449 (14)	0.0826 (17)	0.0272 (13)	0.0033 (13)	0.0124 (12)
C40	0.0576 (13)	0.0498 (13)	0.0448 (12)	0.0278 (11)	0.0139 (10)	0.0160 (10)
C41	0.0560 (13)	0.0470 (13)	0.0480 (13)	0.0279 (11)	0.0078 (11)	0.0135 (10)
C42	0.0650 (15)	0.0743 (17)	0.0573 (15)	0.0386 (14)	0.0113 (12)	0.0117 (13)
C43	0.0835 (19)	0.0774 (18)	0.0542 (15)	0.0437 (16)	0.0008 (14)	0.0027 (13)
C44	0.0768 (18)	0.0536 (15)	0.0696 (17)	0.0253 (14)	−0.0079 (14)	0.0158 (13)
C45	0.0599 (15)	0.0656 (17)	0.0803 (18)	0.0141 (13)	0.0089 (14)	0.0302 (15)
C46	0.0644 (16)	0.0639 (15)	0.0572 (14)	0.0239 (13)	0.0173 (12)	0.0221 (12)
C47	0.096 (2)	0.082 (2)	0.106 (2)	0.0169 (18)	−0.0275 (18)	0.0158 (18)
C48	0.0609 (14)	0.0509 (13)	0.0538 (13)	0.0333 (12)	0.0023 (11)	0.0075 (11)
C49	0.0808 (17)	0.0587 (15)	0.0656 (15)	0.0418 (14)	0.0127 (13)	0.0156 (12)
C50	0.0661 (16)	0.0585 (15)	0.0641 (15)	0.0339 (13)	0.0132 (13)	0.0162 (12)
C51	0.0710 (18)	0.100 (2)	0.0845 (19)	0.0420 (17)	0.0091 (15)	0.0132 (16)
C52	0.076 (2)	0.141 (3)	0.117 (3)	0.062 (2)	0.029 (2)	0.025 (2)
C53	0.107 (3)	0.136 (3)	0.089 (2)	0.072 (2)	0.042 (2)	0.028 (2)
C54	0.101 (2)	0.116 (3)	0.0665 (18)	0.058 (2)	0.0163 (17)	0.0102 (16)
C55	0.0745 (17)	0.099 (2)	0.0676 (17)	0.0487 (16)	0.0117 (15)	0.0125 (15)
C56	0.0560 (14)	0.0507 (13)	0.0566 (15)	0.0250 (11)	0.0038 (12)	0.0082 (11)
C57	0.0790 (17)	0.0708 (17)	0.0755 (16)	0.0488 (15)	0.0207 (14)	0.0098 (13)
C58	0.0792 (19)	0.098 (2)	0.105 (2)	0.0576 (17)	0.0228 (16)	0.0164 (17)

### Geometric parameters (Å, °)

**Table:**

O1—C7	1.248 (3)	C24—C25	1.358 (4)
O2—C27	1.201 (3)	C24—H24	0.9300
O3—C27	1.320 (3)	C25—C26	1.374 (3)
O3—C28	1.457 (3)	C25—H25	0.9300
O4—C36	1.252 (2)	C26—H26	0.9300
O5—C56	1.195 (2)	C28—C29	1.475 (4)
O6—C56	1.327 (2)	C28—H28A	0.9700
O6—C57	1.456 (2)	C28—H28B	0.9700
N1—C7	1.375 (3)	C29—H29A	0.9600
N1—N2	1.401 (2)	C29—H29B	0.9600
N1—C6	1.415 (3)	C29—H29C	0.9600
N2—C9	1.309 (3)	C30—C35	1.374 (3)
N3—C11	1.325 (3)	C30—C31	1.383 (3)
N3—C19	1.449 (3)	C30—H30	0.9300
N3—H3A	0.8600	C31—C32	1.376 (4)
N4—C36	1.373 (2)	C31—H31	0.9300
N4—N5	1.403 (2)	C32—C33	1.364 (4)
N4—C35	1.416 (3)	C32—H32	0.9300
N5—C38	1.310 (2)	C33—C34	1.374 (3)
N6—C40	1.331 (2)	C33—H33	0.9300
N6—C48	1.445 (2)	C34—C35	1.385 (3)
N6—H6	0.8600	C34—H34	0.9300
C1—C6	1.378 (3)	C36—C37	1.433 (3)
C1—C2	1.396 (4)	C37—C40	1.390 (3)
C1—H1	0.9300	C37—C38	1.433 (3)
C2—C3	1.366 (5)	C38—C39	1.483 (3)
C2—H2	0.9300	C39—H39A	0.9600
C3—C4	1.364 (4)	C39—H39B	0.9600
C3—H3	0.9300	C39—H39C	0.9600
C4—C5	1.383 (3)	C40—C41	1.482 (3)
C4—H4	0.9300	C41—C42	1.379 (3)
C5—C6	1.383 (3)	C41—C46	1.383 (3)
C5—H5	0.9300	C42—C43	1.381 (3)
C7—C8	1.443 (3)	C42—H42	0.9300
C8—C11	1.392 (3)	C43—C44	1.373 (3)
C8—C9	1.426 (3)	C43—H43	0.9300
C9—C10	1.492 (3)	C44—C45	1.374 (3)
C10—H10A	0.9600	C44—C47	1.518 (3)
C10—H10B	0.9600	C45—C46	1.375 (3)
C10—H10C	0.9600	C45—H45	0.9300
C11—C12	1.491 (3)	C46—H46	0.9300
C12—C13	1.371 (3)	C47—H47A	0.9600
C12—C17	1.379 (3)	C47—H47B	0.9600
C13—C14	1.384 (3)	C47—H47C	0.9600
C13—H13	0.9300	C48—C56	1.518 (3)
C14—C15	1.370 (3)	C48—C49	1.540 (3)
C14—H14	0.9300	C48—H48	0.9800
C15—C16	1.375 (3)	C49—C50	1.510 (3)
C15—C18	1.521 (3)	C49—H49A	0.9700
C16—C17	1.377 (3)	C49—H49B	0.9700
C16—H16	0.9300	C50—C51	1.372 (3)
C17—H17	0.9300	C50—C55	1.374 (3)
C18—H18A	0.9599	C51—C52	1.378 (4)
C18—H18B	0.9600	C51—H51	0.9300
C18—H18C	0.9600	C52—C53	1.368 (4)
C19—C27	1.512 (3)	C52—H52	0.9300
C19—C20	1.538 (3)	C53—C54	1.350 (4)
C19—H19	0.9800	C53—H53	0.9300
C20—C21	1.506 (3)	C54—C55	1.371 (3)
C20—H20A	0.9700	C54—H54	0.9300
C20—H20B	0.9700	C55—H55	0.9300
C21—C26	1.375 (3)	C57—C58	1.491 (3)
C21—C22	1.378 (3)	C57—H57A	0.9700
C22—C23	1.382 (3)	C57—H57B	0.9700
C22—H22	0.9300	C58—H58A	0.9600
C23—C24	1.373 (3)	C58—H58B	0.9600
C23—H23	0.9300	C58—H58C	0.9600
			
C27—O3—C28	118.0 (2)	C29—C28—H28B	110.2
C56—O6—C57	117.64 (17)	H28A—C28—H28B	108.5
C7—N1—N2	111.98 (17)	C28—C29—H29A	109.5
C7—N1—C6	128.7 (2)	C28—C29—H29B	109.5
N2—N1—C6	119.28 (19)	H29A—C29—H29B	109.5
C9—N2—N1	106.36 (17)	C28—C29—H29C	109.5
C11—N3—C19	128.05 (18)	H29A—C29—H29C	109.5
C11—N3—H3A	116.0	H29B—C29—H29C	109.5
C19—N3—H3A	116.0	C35—C30—C31	119.5 (2)
C36—N4—N5	111.77 (16)	C35—C30—H30	120.2
C36—N4—C35	129.02 (18)	C31—C30—H30	120.2
N5—N4—C35	119.18 (16)	C32—C31—C30	120.3 (3)
C38—N5—N4	106.38 (16)	C32—C31—H31	119.9
C40—N6—C48	127.15 (18)	C30—C31—H31	119.9
C40—N6—H6	116.4	C33—C32—C31	120.0 (3)
C48—N6—H6	116.4	C33—C32—H32	120.0
C6—C1—C2	118.7 (3)	C31—C32—H32	120.0
C6—C1—H1	120.7	C32—C33—C34	120.3 (3)
C2—C1—H1	120.7	C32—C33—H33	119.9
C3—C2—C1	120.7 (3)	C34—C33—H33	119.9
C3—C2—H2	119.7	C33—C34—C35	120.0 (2)
C1—C2—H2	119.7	C33—C34—H34	120.0
C4—C3—C2	120.1 (3)	C35—C34—H34	120.0
C4—C3—H3	119.9	C30—C35—C34	119.9 (2)
C2—C3—H3	119.9	C30—C35—N4	121.19 (19)
C3—C4—C5	120.5 (3)	C34—C35—N4	118.9 (2)
C3—C4—H4	119.7	O4—C36—N4	125.45 (19)
C5—C4—H4	119.7	O4—C36—C37	129.52 (18)
C6—C5—C4	119.4 (3)	N4—C36—C37	105.02 (17)
C6—C5—H5	120.3	C40—C37—C38	132.1 (2)
C4—C5—H5	120.3	C40—C37—C36	122.48 (18)
C1—C6—C5	120.6 (2)	C38—C37—C36	105.47 (17)
C1—C6—N1	119.3 (2)	N5—C38—C37	111.32 (19)
C5—C6—N1	120.1 (2)	N5—C38—C39	118.58 (19)
O1—C7—N1	126.4 (2)	C37—C38—C39	130.0 (2)
O1—C7—C8	129.1 (2)	C38—C39—H39A	109.5
N1—C7—C8	104.5 (2)	C38—C39—H39B	109.5
C11—C8—C9	132.3 (2)	H39A—C39—H39B	109.5
C11—C8—C7	122.2 (2)	C38—C39—H39C	109.5
C9—C8—C7	105.55 (18)	H39A—C39—H39C	109.5
N2—C9—C8	111.57 (19)	H39B—C39—H39C	109.5
N2—C9—C10	118.3 (2)	N6—C40—C37	118.00 (19)
C8—C9—C10	130.1 (2)	N6—C40—C41	117.83 (18)
C9—C10—H10A	109.5	C37—C40—C41	124.17 (18)
C9—C10—H10B	109.5	C42—C41—C46	118.2 (2)
H10A—C10—H10B	109.5	C42—C41—C40	121.3 (2)
C9—C10—H10C	109.5	C46—C41—C40	120.46 (19)
H10A—C10—H10C	109.5	C41—C42—C43	120.4 (2)
H10B—C10—H10C	109.5	C41—C42—H42	119.8
N3—C11—C8	119.18 (19)	C43—C42—H42	119.8
N3—C11—C12	120.08 (18)	C44—C43—C42	121.5 (2)
C8—C11—C12	120.7 (2)	C44—C43—H43	119.2
C13—C12—C17	119.0 (2)	C42—C43—H43	119.2
C13—C12—C11	120.66 (18)	C43—C44—C45	117.6 (2)
C17—C12—C11	120.16 (19)	C43—C44—C47	121.5 (3)
C12—C13—C14	119.9 (2)	C45—C44—C47	120.9 (3)
C12—C13—H13	120.0	C44—C45—C46	121.7 (2)
C14—C13—H13	120.0	C44—C45—H45	119.2
C15—C14—C13	121.8 (2)	C46—C45—H45	119.2
C15—C14—H14	119.1	C45—C46—C41	120.5 (2)
C13—C14—H14	119.1	C45—C46—H46	119.8
C14—C15—C16	117.4 (2)	C41—C46—H46	119.8
C14—C15—C18	121.0 (3)	C44—C47—H47A	109.5
C16—C15—C18	121.7 (2)	C44—C47—H47B	109.5
C15—C16—C17	121.9 (2)	H47A—C47—H47B	109.5
C15—C16—H16	119.1	C44—C47—H47C	109.5
C17—C16—H16	119.1	H47A—C47—H47C	109.5
C16—C17—C12	119.9 (2)	H47B—C47—H47C	109.5
C16—C17—H17	120.0	N6—C48—C56	109.45 (17)
C12—C17—H17	120.0	N6—C48—C49	111.18 (18)
C15—C18—H18A	109.5	C56—C48—C49	107.89 (17)
C15—C18—H18B	109.5	N6—C48—H48	109.4
H18A—C18—H18B	109.5	C56—C48—H48	109.4
C15—C18—H18C	109.5	C49—C48—H48	109.4
H18A—C18—H18C	109.5	C50—C49—C48	114.14 (18)
H18B—C18—H18C	109.5	C50—C49—H49A	108.7
N3—C19—C27	110.08 (19)	C48—C49—H49A	108.7
N3—C19—C20	110.38 (19)	C50—C49—H49B	108.7
C27—C19—C20	108.61 (18)	C48—C49—H49B	108.7
N3—C19—H19	109.2	H49A—C49—H49B	107.6
C27—C19—H19	109.2	C51—C50—C55	117.6 (2)
C20—C19—H19	109.2	C51—C50—C49	121.6 (2)
C21—C20—C19	113.60 (19)	C55—C50—C49	120.8 (2)
C21—C20—H20A	108.8	C50—C51—C52	120.7 (3)
C19—C20—H20A	108.8	C50—C51—H51	119.6
C21—C20—H20B	108.8	C52—C51—H51	119.6
C19—C20—H20B	108.8	C53—C52—C51	120.3 (3)
H20A—C20—H20B	107.7	C53—C52—H52	119.8
C26—C21—C22	118.0 (2)	C51—C52—H52	119.8
C26—C21—C20	120.3 (2)	C54—C53—C52	119.5 (3)
C22—C21—C20	121.7 (2)	C54—C53—H53	120.2
C21—C22—C23	120.4 (2)	C52—C53—H53	120.2
C21—C22—H22	119.8	C53—C54—C55	120.2 (3)
C23—C22—H22	119.8	C53—C54—H54	119.9
C24—C23—C22	120.2 (3)	C55—C54—H54	119.9
C24—C23—H23	119.9	C54—C55—C50	121.6 (2)
C22—C23—H23	119.9	C54—C55—H55	119.2
C25—C24—C23	119.9 (3)	C50—C55—H55	119.2
C25—C24—H24	120.0	O5—C56—O6	125.0 (2)
C23—C24—H24	120.0	O5—C56—C48	124.8 (2)
C24—C25—C26	119.7 (3)	O6—C56—C48	110.16 (18)
C24—C25—H25	120.2	O6—C57—C58	107.41 (19)
C26—C25—H25	120.2	O6—C57—H57A	110.2
C25—C26—C21	121.8 (2)	C58—C57—H57A	110.2
C25—C26—H26	119.1	O6—C57—H57B	110.2
C21—C26—H26	119.1	C58—C57—H57B	110.2
O2—C27—O3	124.7 (2)	H57A—C57—H57B	108.5
O2—C27—C19	124.7 (2)	C57—C58—H58A	109.5
O3—C27—C19	110.4 (2)	C57—C58—H58B	109.5
O3—C28—C29	107.6 (2)	H58A—C58—H58B	109.5
O3—C28—H28A	110.2	C57—C58—H58C	109.5
C29—C28—H28A	110.2	H58A—C58—H58C	109.5
O3—C28—H28B	110.2	H58B—C58—H58C	109.5
			
C7—N1—N2—C9	−0.9 (2)	C20—C19—C27—O3	−77.0 (2)
C6—N1—N2—C9	177.74 (19)	C27—O3—C28—C29	173.4 (2)
C36—N4—N5—C38	0.9 (2)	C35—C30—C31—C32	−0.2 (4)
C35—N4—N5—C38	179.14 (17)	C30—C31—C32—C33	1.4 (4)
C6—C1—C2—C3	0.3 (5)	C31—C32—C33—C34	−1.3 (4)
C1—C2—C3—C4	−0.5 (5)	C32—C33—C34—C35	0.1 (4)
C2—C3—C4—C5	0.5 (5)	C31—C30—C35—C34	−1.0 (3)
C3—C4—C5—C6	−0.3 (5)	C31—C30—C35—N4	177.2 (2)
C2—C1—C6—C5	−0.1 (4)	C33—C34—C35—C30	1.0 (3)
C2—C1—C6—N1	179.3 (2)	C33—C34—C35—N4	−177.2 (2)
C4—C5—C6—C1	0.1 (4)	C36—N4—C35—C30	25.2 (3)
C4—C5—C6—N1	−179.3 (2)	N5—N4—C35—C30	−152.77 (19)
C7—N1—C6—C1	157.5 (2)	C36—N4—C35—C34	−156.7 (2)
N2—N1—C6—C1	−20.9 (3)	N5—N4—C35—C34	25.4 (3)
C7—N1—C6—C5	−23.1 (4)	N5—N4—C36—O4	178.62 (19)
N2—N1—C6—C5	158.5 (2)	C35—N4—C36—O4	0.6 (3)
N2—N1—C7—O1	−179.0 (2)	N5—N4—C36—C37	−1.7 (2)
C6—N1—C7—O1	2.5 (4)	C35—N4—C36—C37	−179.78 (19)
N2—N1—C7—C8	1.7 (2)	O4—C36—C37—C40	1.4 (3)
C6—N1—C7—C8	−176.8 (2)	N4—C36—C37—C40	−178.28 (18)
O1—C7—C8—C11	−2.5 (4)	O4—C36—C37—C38	−178.5 (2)
N1—C7—C8—C11	176.71 (19)	N4—C36—C37—C38	1.9 (2)
O1—C7—C8—C9	179.0 (2)	N4—N5—C38—C37	0.4 (2)
N1—C7—C8—C9	−1.8 (2)	N4—N5—C38—C39	−177.18 (18)
N1—N2—C9—C8	−0.4 (2)	C40—C37—C38—N5	178.7 (2)
N1—N2—C9—C10	177.50 (18)	C36—C37—C38—N5	−1.4 (2)
C11—C8—C9—N2	−176.9 (2)	C40—C37—C38—C39	−4.1 (4)
C7—C8—C9—N2	1.4 (2)	C36—C37—C38—C39	175.8 (2)
C11—C8—C9—C10	5.5 (4)	C48—N6—C40—C37	165.84 (19)
C7—C8—C9—C10	−176.2 (2)	C48—N6—C40—C41	−14.5 (3)
C19—N3—C11—C8	−174.1 (2)	C38—C37—C40—N6	173.7 (2)
C19—N3—C11—C12	4.7 (3)	C36—C37—C40—N6	−6.1 (3)
C9—C8—C11—N3	−176.2 (2)	C38—C37—C40—C41	−6.0 (4)
C7—C8—C11—N3	5.8 (3)	C36—C37—C40—C41	174.20 (18)
C9—C8—C11—C12	5.0 (4)	N6—C40—C41—C42	112.5 (2)
C7—C8—C11—C12	−173.09 (19)	C37—C40—C41—C42	−67.8 (3)
N3—C11—C12—C13	90.7 (3)	N6—C40—C41—C46	−65.4 (3)
C8—C11—C12—C13	−90.5 (3)	C37—C40—C41—C46	114.3 (2)
N3—C11—C12—C17	−93.4 (3)	C46—C41—C42—C43	0.4 (3)
C8—C11—C12—C17	85.4 (3)	C40—C41—C42—C43	−177.6 (2)
C17—C12—C13—C14	−1.9 (3)	C41—C42—C43—C44	0.1 (4)
C11—C12—C13—C14	174.0 (2)	C42—C43—C44—C45	−0.9 (4)
C12—C13—C14—C15	0.0 (4)	C42—C43—C44—C47	179.4 (2)
C13—C14—C15—C16	1.3 (4)	C43—C44—C45—C46	1.3 (4)
C13—C14—C15—C18	179.7 (2)	C47—C44—C45—C46	−179.0 (2)
C14—C15—C16—C17	−0.7 (4)	C44—C45—C46—C41	−0.9 (4)
C18—C15—C16—C17	−179.1 (3)	C42—C41—C46—C45	0.0 (3)
C15—C16—C17—C12	−1.2 (4)	C40—C41—C46—C45	178.0 (2)
C13—C12—C17—C16	2.5 (3)	C40—N6—C48—C56	134.3 (2)
C11—C12—C17—C16	−173.5 (2)	C40—N6—C48—C49	−106.6 (2)
C11—N3—C19—C27	−120.7 (2)	N6—C48—C49—C50	58.3 (2)
C11—N3—C19—C20	119.4 (2)	C56—C48—C49—C50	178.36 (19)
N3—C19—C20—C21	−68.9 (2)	C48—C49—C50—C51	−95.1 (3)
C27—C19—C20—C21	170.4 (2)	C48—C49—C50—C55	84.4 (3)
C19—C20—C21—C26	−94.8 (3)	C55—C50—C51—C52	0.8 (4)
C19—C20—C21—C22	84.4 (3)	C49—C50—C51—C52	−179.7 (3)
C26—C21—C22—C23	−0.6 (4)	C50—C51—C52—C53	0.4 (5)
C20—C21—C22—C23	−179.8 (2)	C51—C52—C53—C54	−1.4 (5)
C21—C22—C23—C24	−0.2 (4)	C52—C53—C54—C55	1.3 (5)
C22—C23—C24—C25	0.6 (4)	C53—C54—C55—C50	−0.2 (5)
C23—C24—C25—C26	−0.3 (4)	C51—C50—C55—C54	−0.9 (4)
C24—C25—C26—C21	−0.5 (4)	C49—C50—C55—C54	179.5 (2)
C22—C21—C26—C25	1.0 (4)	C57—O6—C56—O5	4.4 (3)
C20—C21—C26—C25	−179.9 (2)	C57—O6—C56—C48	−172.72 (19)
C28—O3—C27—O2	−1.8 (4)	N6—C48—C56—O5	21.9 (3)
C28—O3—C27—C19	173.7 (2)	C49—C48—C56—O5	−99.2 (3)
N3—C19—C27—O2	−22.5 (3)	N6—C48—C56—O6	−160.91 (17)
C20—C19—C27—O2	98.5 (3)	C49—C48—C56—O6	78.0 (2)
N3—C19—C27—O3	162.01 (19)	C56—O6—C57—C58	−171.0 (2)

### Hydrogen-bond geometry (Å, °)

**Table:**

Cg6 is the centroid of C30–C35 ring.

**Table:**

*D*—H···*A*	*D*—H	H···*A*	*D*···*A*	*D*—H···*A*
N3—H3A···O1	0.86	2.01	2.713 (2)	138
N6—H6···O4	0.86	2.03	2.698 (2)	133
C16—H16···O6^i^	0.93	2.51	3.397 (3)	161
C28—H28B···O1^ii^	0.97	2.44	3.357 (3)	157
C45—H45···Cg6^iii^	0.93	2.77	3.449 (3)	130
C57—H57B···Cg6^iv^	0.96	2.78	3.663 (3)	152

Symmetry codes: (i) *x*+1, *y*, *z*; (ii) −*x*+1, −*y*+2, −*z*+1; (iii) −*x*, −*y*+1, −*z*+2; (iv) *x*−1, *y*, *z*.
